# Supplementary material for: Targeting Pharmacokinetic Drug Resistance in Acute Myeloid Leukemia Cells with CDK4/6 Inhibitors
Source: Cancers (Basel). 2020 Jun 16;12(6):1596. doi: 10.3390/cancers12061596 (PMC7352292; doi:10.3390/cancers12061596)
Supplement: Supplementary file 1 [file cancers-12-01596-s001.pdf]

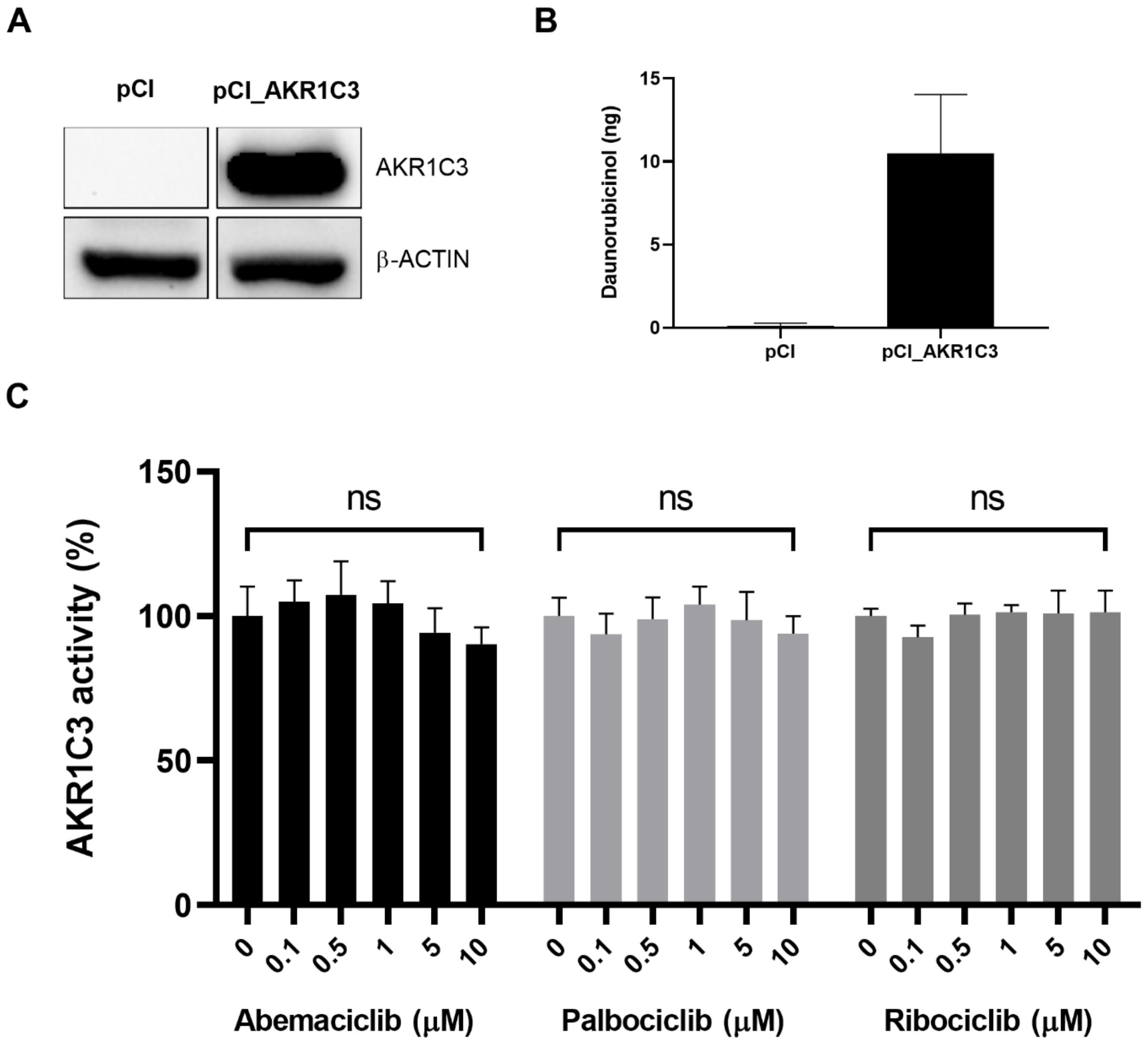

Figure S1. Effect of CDK4/6 inhibitors on reducing activity of HCT116 cells expressing AKR1C3. The expression of AKR1C3 and the intracellular metabolism of daunorubicin was compared to cells transfected with control pCI by western blotting (**A**) and HPLC quantification of daunorubicinol (**B**), respectively. HCT116 cells expressing AKR1C3 were exposed to a range of concentrations of the tested drugs and 1 μM daunorubicin. After 4 h, daunorubicinol production was measured by ultra-high-performance liquid chromatography (**C**). The columns represent mean ± SD from three independent experiments. Data was analyzed by one-way *ANOVA* with Dunnett's multiple comparison test.

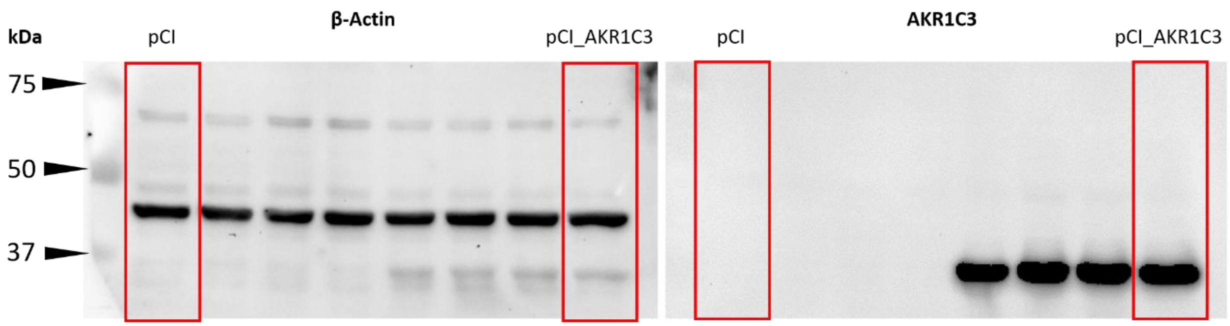

Figure S2. Full western blotting image showing expression of AKR1C3 in transfected cells and lack of this band in the empty plasmid control HCT116 cells (relevant samples used for this study are highlighted in red). The same level of expression of β-Actin is maintained in both cell lines.
